# Isolated MLH1 Loss by Immunohistochemistry Because of Benign Germline *MLH1* Polymorphisms

**DOI:** 10.1200/PO.22.00227

**Published:** 2022-08-31

**Authors:** Dustin E. Bosch, Matthew M. Yeh, Stephen J. Salipante, Angela Jacobson, Stacey A. Cohen, Eric Q. Konnick, Vera A. Paulson

**Affiliations:** ^1^ Department of Laboratory Medicine and Pathology, University of Washington School of Medicine, Seattle, WA; ^2^ Department of Pathology, Roy J. and Lucille A. Carver College of Medicine, University of Iowa, Iowa City, IA; ^3^ Division of Medical Oncology, Department of Medicine, University of Washington School of Medicine, Seattle, WA; ^4^ Clinical Research Division, Fred Hutchinson Cancer Research Center, Seattle, WA

## Abstract

Mismatch repair (MMR) immunohistochemistry (IHC) is frequently used to inform prognosis, select (immuno-)therapy, and identify patients for heritable cancer syndrome testing. However, false-negative and false-positive MMR IHC interpretations have been described.

**MATERIALS AND METHODS:**

Following identification of discordant MMR IHC and DNA-based microsatellite instability testing in a patient with colorectal carcinoma, we retrospectively reviewed institutional archives to identify patient samples with similar discrepancies.

**RESULTS:**

We report a patient with metastatic colorectal carcinoma who initially received immunotherapy on the basis of apparent isolated loss of MLH1 by IHC; notably, *MLH1* promoter hypermethylation was negative. Subsequent evaluation of neoplastic tissue on a DNA-based targeted next-generation sequencing panel demonstrated microsatellite stability, low tumor mutational burden, and a benign MLH1 variant, MLH1 p.V384D, accompanied by loss of heterozygosity. The constellation of findings and repeat MLH1 IHC demonstrating retained expression using a different antibody-clone, supported reclassification of the neoplasm as MMR-proficient. Immunotherapy was discontinued, and cytotoxic chemotherapy was initiated. This index case of apparent discordance between MMR IHC and DNA-based microsatellite instability prompted a retrospective review of institutional archives to identify patient samples with similar discrepancies. Further evaluation of neoplasms harboring MLH1 p.V384D with loss of heterozygosity revealed systematic antibody-dependent interference. The review also identified a second IHC-interference candidate, MLH1 p.A441T.

**CONCLUSION:**

This study confirms that rare germline polymorphisms can result in incorrect IHC results, potentially affecting selection of optimal therapy and the decision to pursue germline testing. This case further highlights the need for expert molecular pathologic review and communication between clinical and molecular oncology teams.

## INTRODUCTION

Mismatch repair (MMR) proteins MLH1, PMS2, MSH2, and MSH6 recognize and correct errors caused by DNA polymerase during replication.^1^ Loss-of-function variants in one of these four proteins result in the accumulation of single-nucleotide variants, insertions, and deletions, particularly in microsatellites, repetitive regions of DNA.^1,2^ Tumors with this phenotype are termed microsatellite unstable (MSI) or MMR-deficient (dMMR); those that retain the ability to correct errors are microsatellite stable (MSS) or MMR-proficient. MMR/MSI status can be assessed using two methodologies: (1) immunohistochemistry (IHC) or (2) molecular MSI testing of extracted DNA, either by polymerase chain reaction (PCR) of standard microsatellite loci (eg, Bethesda or pentaplex panels) or by next-generation sequencing (NGS).^3-5^ NGS methods have potential added benefits of detecting mutations in MMR genes and/or assessing tumor mutational burden (TMB), if appropriately validated.^6^ Concordance between methodologies to detect dMMR status are high, generally > 90%; however, discrepancies do arise.^7,8^ These inconsistencies may result from preanalytical variables, including low tumor cellularity, neoadjuvant treatment, improper fixatives, tumor heterogeneity, functional nonsynonymous mutations, or rarely germline polymorphisms.^9,10^ False-positive MMR IHC has also been described in neoplasms with high mutational burden and *POLE* mutations.^11^

**CONTEXT**

**Key Objective**
Mismatch repair protein immunohistochemistry (IHC) is increasingly used to inform therapy selection, as well as heritable cancer syndrome testing. This case series evaluated causes of discrepancy between mismatch repair IHC and microsatellite instability testing by next-generation sequencing.
**Knowledge Generated**
Two uncommon variants of MLH1, p.V384D and p.A441T, were found to interfere with MLH1 immunohistochemistry, producing the appearance of lost expression, which conflicted with negative microsatellite instability testing. IHC interference was associated with loss of heterozygosity at the *MLH1* locus and was anti-MLH1 antibody clone-dependent (G168-15). In the index case, MLH1 IHC interference led to unnecessary germline genetic testing and affected selection for immunotherapy.
**Relevance**
Rare germline polymorphisms can result in incorrect IHC results, potentially affecting selection of optimal therapy and the decision to pursue germline testing. This case further highlights the need for expert molecular pathologic review and communication between clinical and molecular oncology teams.

MMR IHC and MSI testing have long been used for colorectal and endometrial carcinomas as a screening test for inherited deleterious alterations in MMR genes, which results in Lynch syndrome, previously called hereditary nonpolyposis colorectal cancer (HNPCC), and accounts for approximately 5% of colorectal carcinomas (CRCs).^12^ Patients with Lynch syndrome are also at increased risk for neoplasms of the endometrium, upper gastrointestinal tract, pancreaticobiliary system, urinary tract, prostate, ovaries, and brain.^12^

Somatic dMMR and MSI are also encountered in a variety of neoplasms.^1^ For instance, approximately 15% of sporadic colon carcinomas are dMMR, most commonly because of hypermethylation of the *MLH1* promoter or double somatic mutations in *MLH1* or other MMR genes.^12,13^ MMR status in sporadic neoplasms may also have prognostic significance, as exemplified by longer overall and disease-free survival in patients with dMMR CRC.^14^

Additionally, dMMR/MSI is emerging as an important therapeutic marker, most prominently in predicting response to immune checkpoint inhibitors.^15^ Current evidence supports use of immune checkpoint inhibitors as first-line therapy for advanced dMMR or MSI CRC.^15^ Notably, pembrolizumab is US Food and Drug Administration–approved for any dMMR/MSI neoplasms, regardless of histologic type or site of origin.^16,17^ As a result, laboratory testing for MMR deficiency is increasingly performed to help direct treatment decisions.

Given the importance of MMR/MSI status in selecting patients for additional germline testing, and its role in prognostication and therapeutic selection, pathologists and clinicians should understand factors that might result in improper dMMR classification. We describe a case of false loss of MMR IHC in a patient with metastatic CRC, caused by IHC interference because of a rare benign germline polymorphism in *MLH1* with loss of heterozygosity (LOH). Further investigation identified a series of systematic false loss of MLH1 IHC in a series of clinical cases because of MMR IHC interference. These findings highlight the utility of a comprehensive approach in determining MMR status, and integrating evaluation of MMR genes with assessment of MSI and TMB, with expert molecular pathologist interpretation.

## MATERIALS AND METHODS

### Case Selection and Clinicopathologic Analysis

A morphomolecular discrepancy was identified in a clinical case submitted for NGS as part of an institutional review board–approved study designed to comprehensively evaluate gastrointestinal malignancies for single nucleotide variants, indels, fusions, and MSI/TMB status. This patient (indicated as case 1 in Table 1) provided informed consent for publication. Following institutional review board protocol approval at the University of Washington, cases were selected by searching institutional laboratory information systems (PowerPath, University of Washington Medical Center (UWMC) genetics database) for neoplastic specimens with concurrent MMR IHC, MSI testing, and/or MMR gene sequencing. This search yielded two additional cases with discrepancy between MLH1 IHC and microsatellite status, and another with a candidate MLH1 IHC-interference variant and LOH (four cases total). All available histologic slides, immunohistochemical stains, and diagnoses were reviewed by board-certified anatomic pathologists (M.M.Y. and D.E.B.), and molecular data were reviewed by board-certified molecular pathologists (E.Q.K. and V.A.P.). Clinicopathologic demographics, including age, sex, diagnoses, and treatment data, were gathered from the electronic health record.

**TABLE 1.**
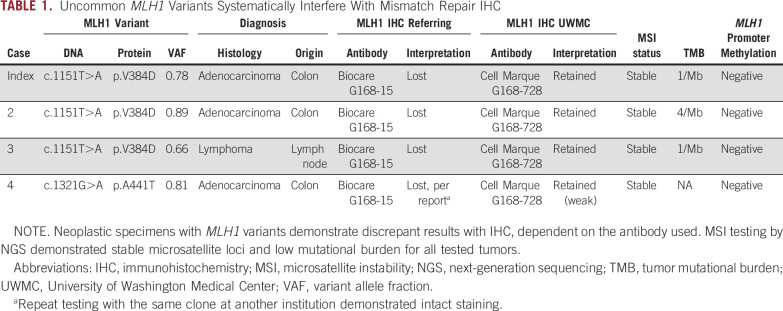
Uncommon *MLH1* Variants Systematically Interfere With Mismatch Repair IHC

### MMR IHC

MMR IHC was performed at UWMC and referring institutions; laboratories and antibodies used are listed in Table 1. At UWMC, IHC was performed on 4-μm thick unstained slides from formalin-fixed paraffin-embedded (FFPE) tissue blocks using an automated platform (BOND-III; Leica Microsystems, Buffalo Grove, IL). Following deparaffinization and rehydration, slides were rinsed and incubated with the primary antibody, washed in buffer, followed by incubation with a peroxidase-labeled polymer (BOND Polymer Refine; Leica). Bound antibody was localized via a peroxidase reaction with 3,3′-diaminobenzidine tetrahydrochloride (DAB+; Dako, Carpinteria, CA) as chromogen. Slides were washed in water, counterstained using hematoxylin, dehydrated, and mounted. Positive controls were performed to evaluate for appropriate staining. All immunohistochemical testing was performed in Clinical Laboratory Improvement Amendments–certified clinical laboratories.

### Targeted NGS

Molecular characterization was performed on one of two DNA-based targeted next-generation sequencing panels, as previously described.^18,19^ In brief, DNA was extracted from FFPE tissue using the Qiagen GeneRead DNA FFPE Kit (Qiagen, Valencia, CA) before shearing and library preparation using KAPA HyperPrep reagents (Roche, Wilmington, MA). Prepared libraries were hybridized to a set of custom probes designed to target panels of genes chosen for their relevance in cancer diagnosis, prognosis, and/or treatment (UW-OncoPlex version 6, which targets 340 genes) or their importance in cancer-susceptibility (BROCA, which targets 69 genes). Libraries were then sequenced on Illumina NextSeq500 and HiSeq2500 systems (Illumina, San Diego, CA), and sequences were processed through an automated, custom-designed bioinformatics pipeline developed by the University of Washington NGS Laboratory and Analytics group before analysis by board-certified molecular pathologists (E.Q.K. and V.A.P.). In addition to identifying single-nucleotide variants, insertions and deletions, fusions, and copy-number alterations, these assays detect microsatellite instability and TMB (OPXv6 only).^2^

### MLH1 Methylation

After sodium bisulfite conversion using EZ DNA Methylation-Lightning Kit (Zymo Research, Irvine CA, Cat. No. D5030), tumor DNA was amplified using fluorescence-based, real-time quantitative PCR, as previously described.^20^ In brief, the promoter region of *MLH1* was amplified using methyl-specific CG-specific PCR primers flanking an oligonucleotide probe with a 5ʹ fluorescent reporter dye (6-FAM) and a 3ʹ quencher dye (BHQ); the housekeeping gene *COL2A1* was amplified for normalization of DNA input using the ViiA 7 Real-Time PCR Instrument (Thermo Fisher Scientific, Waltham, MA), and the results were evaluated by a molecular pathologist (E.Q.K.).

### Consent for Publication

The patient discussed as the index case provided informed consent for publication.

## RESULTS

**FIG 1.**
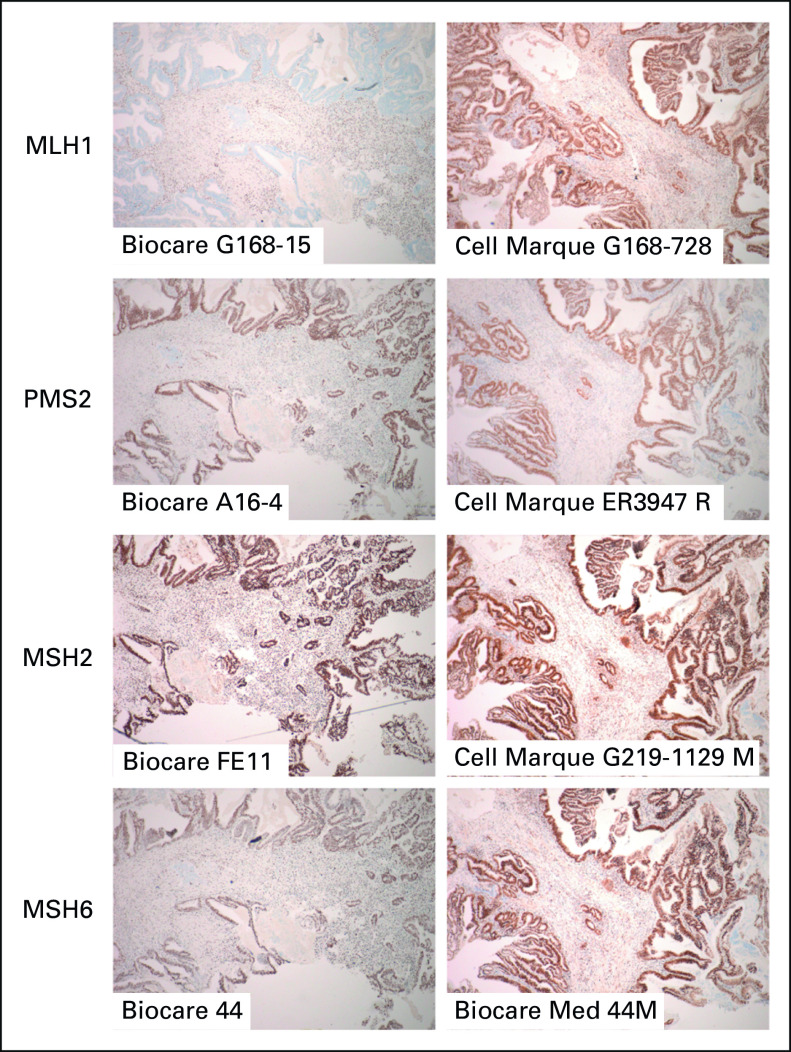
Mismatch repair protein IHC demonstrates antibody-dependent interference by MLH1 p.V384D. Micrographs of IHC results are shown for the index case, performed at two institutions with different antibodies. MLH1 appears lost with G168-15, but expression is intact with G168-728. IHC, immunohistochemistry.

The index case prompting this series was a 50-year-old male patient, who presented to an outside institution with anorectal bleeding. Colonoscopy demonstrated a rectal mass, and biopsy revealed an invasive adenocarcinoma arising in an adenoma. MMR IHC was performed at the outside institution and showed loss of MLH1 expression (Biocare G168-15 antibody) with intact PMS2, MSH2, and MSH6 by IHC (Fig 1). Subsequent *MLH1* promoter hypermethylation testing was negative. Staging imaging revealed stage IV disease with direct local invasion of a seminal vesicle and multiple enlarged regional and nonregional lymph nodes. Upon referral to our institution, initiation with systemic therapy was recommended by the multidisciplinary team on the basis of the stage of disease, with the intent to complete a short course of systemic chemotherapy, followed by chemoradiation to the rectal primary and all nodal disease (including the M1a nonregional nodes) and then resection of the primary. On the basis of the apparent dMMR status, treatment with pembrolizumab was initiated. As part of a research protocol to uniformly perform panel-based testing, the initial colonoscopy biopsy was tested for MSI by NGS, revealing MSS status.^2^ In addition to revealing low TMB (1 mutation/megabase), the panel also identified *ERBB2* amplification (for a complete molecular profile of the tumor, see the Data Supplement). Germline genetic testing was concurrently sent, and ultimately resulted as negative for pathogenic *MLH1* variants, although a variant of uncertain significance was noted in *PMS2*.

Given the discrepancy in this case between NGS sequencing results and MMR IHC, rare germline polymorphisms in the MMR genes were further reviewed. This identified an *MLH1* polymorphism (p.V384D, NM_000249.3:c.1151T>A) accompanied by LOH (variant allele fraction [VAF] 0.78). This *MLH1* variant, which has been classified as NOT pathogenic on the basis of criteria developed by the InSiGHT Mutation Interpretation Committee, has previously been reported in the literature in association with MLH1 IHC loss.^21^ IHC at our institution using a different antibody clone (Cell Marque G168-728) revealed intact expression of MLH1 (Fig 1), and the neoplasm was subsequently reclassified as MMR-proficient. For the patient of interest, immunotherapy was discontinued upon recognition of the MMR-proficient status, and treatment with infusional fluorouracil, leucovorin, and oxaliplatin and bevacizumab was initiated. Restaging was not performed after the single cycle of pembrolizumab, but there was minimal change in the carcinoembryonic antigen (109-85 ng/mL) during that first cycle, whereas a more dramatic decrease (85-4 ng/mL) in the subsequent 2 months of cytotoxic chemotherapy was observed, suggesting minimal clinical benefit with immunotherapy. The patient subsequently underwent a low anterior resection with diverting loop ileostomy. Histologic examination of the resection specimen revealed only a single focus (0.3 cm) of moderately differentiated adenocarcinoma with no evidence of lymphatic or perineurial invasion; all evaluated lymph nodes were negative. The patient continues to do well on therapy more than 6 months after his surgical resection, with negative imaging and no evidence of minimal residual disease using the Signatera ctDNA assay.

Two additional neoplastic cases harboring the MLH1 p.V384D variant accompanied by LOH were identified from institutional archives. Both cases were MSS with low mutational burdens (Table 1). Similar to the index case, MLH1 expression was not detected using the MLH1 G168-15 antibody, but was retained using the G168-728 antibody (Table 1). Neither of these patients received immunotherapy or underwent germline genetic testing per medical record review.

To identify other nonpathogenic variants with potential MMR IHC interference, we queried our anatomic pathology and molecular databases for neoplastic cases with isolated MMR protein loss and discordant MSS status by NGS testing. One case with apparent loss of MLH1 by IHC at the referring institution but MSS by NGS was found to harbor a different benign germline polymorphism, MLH1 p.A441T, with associated LOH in neoplastic tissue. MLH1 IHC with the G168-728 antibody at our institution demonstrated weak, but intact (retained), expression (Table 1). Treatment course for this patient is unknown (reference laboratory testing only).

## DISCUSSION

With the increasing utility of MSI testing for clinical and therapeutic decision making, valid and reliable testing is paramount. Herein, we report two germline *MLH1* variants, MLH1 p.V384D and MLH1 p.A441T, which appear to interfere with MMR IHC, resulting in isolated false loss of MLH1 expression.^22^ The former *MLH1* variant is relatively common in some populations, with a maximum allele frequency of up to 0.03, whereas the latter only occurs at a maximum allele frequency of 0.001.^23^ The frequency of expected IHC interference by combined presence of a MLH1 p.V384D or p.A441T and LOH is difficult to estimate for several reasons: frequencies of these polymorphisms vary by population, LOH is not uniformly measured or reported in databases, and quantification of the baseline frequency of LOH at the MLH1 locus is confounded by MSI-high neoplasms. In a 7-year period of testing neoplastic tissues with NGS at our institution, MLH1 p.V384D was detected 123 times and associated with possible LOH (VAF > 0.6) in 11 cases (approximately 9%). Multiplying this value by the global minor allele frequency for MLH1 p.V384D (0.00519)^22^ crudely estimates this combination of events may occur in 1 of every 2,000 cases. Although this number is only an estimate on the basis of limited data, it does suggest that this type of event is uncommon.

Substitution of the neutral hydrophobic amino acid valine at codon 384 for negatively charged aspartic acid is not a conservative change, but this alteration occurs in a poorly conserved region of *MLH1* outside of its known functional domains.^24,25^ Prior studies investigating the functional consequences of the MLH1 p.V384D variant suggested mildly reduced coimmunoprecipitation with PMS2 and weakened β-galactosidase activity in a yeast two-hybrid assay with PMS2.^26^ However, Takahashi et al^27^ reported the MMR activity of both the p.V384D and p.A441T variants in yeast and in vitro MMR assays to be within their assay's normal limits (defined as 60% or higher). Previous publications describing focal MLH1 loss of expression in association with MLH1 p.V384D did not show increased microsatellite instability, and TMB remained low.^28^ These findings are concordant with our own, in which tumors harboring p.V384D were MSS with a low TMB, despite associated LOH. Whether MLH1 p.V384D is associated with carcinoma risk, despite the lack of contribution to MSI status, remains controversial. Multiple case-control style studies have reported enrichment of p.V384D among patients with colon cancer.^24,25^ How or if this germline allele may contribute to carcinogenesis is unclear, given the nonassociation with familial cancer syndromes, lack of consistent second hit variants in the neoplasms, and lack of association with microsatellite instability. Both MLH1 variants, p.V384D and p.A441T, have been classified as benign (ClinVar IDs: 41632 and 89696).^22^ Regardless, this uncommon germline variant is not associated with Lynch syndrome or microsatellite instability. Thus, it still constitutes a false-positive interference with MMR IHC in the context of this study.

IHC for MMR proteins is commonly used as a screening test for Lynch syndrome, typically in combination with *BRAF* genetic testing and/or *MLH1* promoter hypermethylation testing to exclude the majority of somatic deleterious variants.^7^ Thus, neoplasms with loss of MLH1 by IHC, negative for *MLH1* promoter hypermethylation and/or negative for *BRAF* p.V600E, are recommended for germline MMR gene testing. Following this pathway, our index case was negative for *MLH1* promoter hypermethylation, prompting referral for medical genetics to determine the etiology of his MLH1 loss. Tumor NGS was also performed as part of a research protocol to uniformly perform panel-based testing, including evaluation of MSI by NGS. The apparent discrepancy between MLH1 IHC and MSI testing by NGS was identified and repeat MLH1 IHC with a different antibody demonstrated intact expression.

Given that MLH1 loss was demonstrably dependent on the antibodies used for MLH1 IHC, we hypothesize that these specific variants disrupt an epitope recognized by a subset of anti-MLH1 antibodies. Since similar IHC patterns were observed for both MLH1 p.V384D and MLH1 p.A441T, ie, apparent loss with antibodies other than G168-728, we predict that these two amino acids contribute to a common epitope. To our knowledge, epitope mapping has not been published for the antibodies used in this study and protein structural data for MLH1 are unavailable for the region spanning p.V384—A441, precluding examination of their spatial relationship.^29,30^ On the basis of prior studies using G168-728, the MLH1 region containing an epitope can be narrowed to amino acids 321-505.^31,32^ Exon 16 truncation mutations of MLH1 exhibit retained immunoreactivity with G168-15, indicating that an epitope lies in the amino acid range 1-632.^33^ Specific epitopes for other MLH1 antibody clones commonly used in clinical IHC, such as M1 (Ventana) and ES05, are not published. Additional studies are necessary to determine whether p.V384D and p.A441T interfere with these antibody clones. The IHC results of case 4, however (Table 1), imply that MLH1 IHC interlaboratory discrepancies are not fully explained by differences in antibody clones. Despite using the same G168-15 antibody, IHC at two different institutions reported conflicting results (intact *v* lost) during the evaluation of MLH1 IHC for case 4. Alternative explanations for differential detection of these MLH1 variants by IHC include differences in other aspects of MMR IHC tests across laboratories, such as antigen-retrieval approaches, binding conditions, or antibody titer, which may conceivably result in variant-specific loss of MLH1 detection. Ultimately, whether the isolated MLH1 loss observed at the referring institution was attributable to IHC interference or one of the alternative explanations, our findings confirmed that germline evaluation for HNPCC in this patient was unnecessary.

Perhaps a more compelling need to understand limitations of MMR IHC is its emerging use in diverse neoplasms as an indication for immune checkpoint inhibitor therapy.^15-17^ Lack of adequate MMR in carcinoma cells predicts response to immunotherapy, and there is generally high concordance among MMR IHC, PCR-based MSI testing, and NGS-based MSI testing in detecting dMMR.^7^ However, false-positive results in any selected modality may result in suboptimal efficacy of immune checkpoint inhibitors and less therapeutic benefit than standard chemotherapies such as infusional fluorouracil, leucovorin, and oxaliplatin for CRCs.^15,34^ MSI testing by NGS, particularly in the context of panel testing, has the advantage of confirming MSI status with simultaneous detection of MMR gene variants and assessment of TMB. However, cost-effective NGS-based MSI testing is not uniformly available. For those institutions where such testing is unavailable, paired testing with PCR-based MSI may be a useful adjunct to MMR IHC when the underlying etiology of IHC loss is unclear or atypical, although MSI PCR may have limitations in noncolorectal cancers.^35^ In the setting of a benign germline variant that causes false loss on MMR IHC testing, the expected pattern would be isolated loss of an MMR protein expression and discordant MSS status. Reflexive testing on a more comprehensive panel might then be advised to determine the source of the discrepancy. Interference with MLH1 IHC by p.V384 and/or p.A441T may be detected using MSS samples with known variants and LOH. Another strategy to mitigate the risk of uncommon variant interference with MLH1 IHC is to maintain a second MLH1 antibody clone for additional testing in cases with isolated loss of MLH1 and/or suspicion of interference.

In summary, we present a case series identifying two rare germline polymorphisms that result in false loss of MLH1 by IHC. In at least one case, this finding led to initial selection of suboptimal therapy and pursuit of unnecessary germline genetic testing. Additional interference variants will likely be identified as MMR/MSI evaluation increases in frequency, given the role in this tumor-agnostic biomarker in selecting patients for possible benefit from immunotherapies,^36^ and soon-to-be-published College of American Pathologists /ASCO guidelines indicating MMR IHC as the recommended laboratory method for assessing dMMR status for immunotherapy selection.^37^ Our findings in these cases highlight the advantages of a comprehensive and integrative approach to determining MMR status, one that integrates expert interpretation and evaluation of MSI, MMR IHC, and TMB status in the context of germline and somatic assessment of MMR gene sequences.
